# miR-505 suppresses prostate cancer progression by targeting NRCAM

**DOI:** 10.3892/or.2020.7696

**Published:** 2020-07-17

**Authors:** Xiao-Hui Ling, Hao Fu, Zhi-Yun Chen, Jian-Ming Lu, Yang-Jia Zhuo, Jia-Hong Chen, Wei-De Zhong, Zhenyu Jia

Oncol Rep 42: 991-1004, 2019; DOI: 10.3892/or.2019.7231

Subsequently to the publication of this article, one of the corresponding authors, Dr Wei-Di Zhong, has realized that the information presented in the box for correspondence for him was incorrect. Although Dr Zhong is correctly shown as having three affiliation addresses in the paper, the address affiliation listed first on the paper should have been presented as the address for correspondence, not the second one. Therefore, the authors affiliation information should have appeared as follows (changes are highlighted in bold):

Correspondence to: Dr Wei- Di Zhong, **Guangdong Provincial Institute of Nephrology, Southern Medical University, Guangzhou, Guangdong 510515, P.R. China**

Dr Zhong deeply regrets his oversight in this regard, and apologizes for any inconvenience caused.

